# Supplementary material for: A Comprehensive Assessment of Ultraviolet-Radiation-Induced Mutations in Flammulina filiformis Using Whole-Genome Resequencing
Source: J Fungi (Basel). 2024 Mar 20;10(3):228. doi: 10.3390/jof10030228 (PMC10971301; doi:10.3390/jof10030228)
Supplement: Supplementary file 1 [file jof-10-00228-s001.zip › Supplementary Material S8/KEGG annotation/out/64381550635650.os/KO/out_map/map04520.html]

KEGG PATHWAY: Adherens junction - Reference pathway


|  |  |
| --- | --- |
| **Adherens junction - Reference pathway** |  |

[
Pathway menu
| Organism menu
| Pathway entry
| Show description
| User data mapping
]

Cell-cell adherens junctions (AJs), the most common type of intercellular adhesions, are important for maintaining tissue architecture and cell polarity and can limit cell movement and proliferation. At AJs, E-cadherin serves as an essential cell adhesion molecules (CAMs). The cytoplasmic tail binds beta-catenin, which in turn binds alpha-catenin. Alpha-catenin is associated with F-actin bundles directly and indirectly. The integrity of the cadherin-catenin complex is negatively regulated by phosphorylation of beta-catenin by receptor tyrosine kinases (RTKs) and cytoplasmic tyrosine kinases (Fer, Fyn, Yes, and Src), which leads to dissociation of the cadherin-catenin complex. Integrity of this complex is positively regulated by beta -catenin phosphorylation by casein kinase II, and dephosphorylation by protein tyrosine phosphatases. Changes in the phosphorylation state of beta-catenin affect cell-cell adhesion, cell migration and the level of signaling beta-catenin. Wnt signaling acts as a positive regulator of beta-catenin by inhibiting beta-catenin degradation, which stabilizes beta-catenin, and causes its accumulation. Cadherin may acts as a negative regulator of signaling beta-catenin as it binds beta-catenin at the cell surface and thereby sequesters it from the nucleus. Nectins also function as CAMs at AJs, but are more highly concentrated at AJs than E-cadherin. Nectins transduce signals through Cdc42 and Rac, which reorganize the actin cytoskeleton, regulate the formation of AJs, and strengthen cell-cell adhesion.

|  |  |
| --- | --- |
| Reference pathway | 100% |
